# Socio-Demographic Determinants of Diet Quality in Australian Adults Using the Validated Healthy Eating Index for Australian Adults (HEIFA-2013)

**DOI:** 10.3390/healthcare5010007

**Published:** 2017-02-04

**Authors:** Amanda Grech, Zhixian Sui, Hong Ying Siu, Miaobing Zheng, Margaret Allman-Farinelli, Anna Rangan

**Affiliations:** Nutrition and Dietetics Group, School of Life and Environmental Sciences, Charles Perkins Centre, The University of Sydney, Sydney, NSW 2006, Australia; zhixian.sui@sydney.edu.au (Z.S.); hsiu6708@uni.sydney.edu.au (H.Y.S.); miaobing.zheng@sydney.edu.au (M.Z.); margaret.allman-farinelli@sydney.edu.au (M.A.-F.); anna.rangan@sydney.edu.au (A.R.)

**Keywords:** diet quality, diet quality index, nutrition, 24-hour recall

## Abstract

Diet quality indices have been shown to predict cardiovascular disease, cancer, Type 2 Diabetes, obesity and all-cause mortality. This study aimed to determine the socio-demographics of Australian adults with poor diet quality. Diet quality was assessed for participants of the 2011–2012 National Nutrition and Physical Activity Survey aged 18 years or above (*n* = 9435), with the validated 11-component Healthy Eating Index for Australians (HEIFA-2013), based on the 2013 Australian Dietary Guidelines. Differences in scores by demographics (ANOVA) and regression models for associations between the HEIFA-2013 score and demographic characteristics were conducted. The mean (SD) HEIFA-2013 score was 45.5 (14.7) out of 100 due to poor intakes of vegetables, fruit, grains, dairy and fat and high intakes of added sugar, sodium and discretionary foods. Lower mean HEIFA-2013 scores (SD) were found for males 43.3 (14.7), young-adults 41.6 (14.2) obese 44.1 (14.3), smokers 40.0 (14.2), low socio-economic status 43.7 (14.9) and Australian country-of-birth 44.2 (14.6) (*p* < 0.05). The overall diet quality of the Australian population is poor and targeted interventions for young-adults, males, obese and those with lower socio-economic status are recommended.

## 1. Introduction

Internationally, dietary guidelines provide recommendations for optimal dietary patterns for health that include a variety of fruit, vegetables, lean meat and alternatives, low-fat dairy and whole grains and are rich in unsaturated fats while minimising deleterious nutrients such as added sugars, saturated fat, alcohol and sodium [1,2,3,4]. Such dietary patterns have been proven to reduce the risk of non-communicable diseases such as Type 2 Diabetes, cardiovascular disease (CVD), some cancers, osteoporosis, and dental disease, and also help to maintain a healthy body weight [2,3]. However, increases in non-communicable diseases in recent decades have been well documented [5,6]. Populations of lower socio-economic status are burdened with a higher incidence of these diseases [7,8]. Understanding the dietary patterns of the population can provide insight into how diet may be contributing to these diseases and identify sub-populations requiring tailored interventions.

A high quality or healthy diet is increasingly understood to be due to the synergistic interaction between nutrients and other food components rather than the action of individual foods or nutrients [9,10]. Diet quality indices have been created to allow all foods consumed to be considered simultaneously, as well as accounting for nutrients and the variety of foods consumed within food groups [11,12]. Higher diet quality scores have been shown to be predictive of reduced risk from all-cause mortality, a lower incidence and mortality from CVD, cancer and reduced risk of obesity and Type 2 Diabetes [13,14,15,16].

The Healthy Eating Index for Australians (HEIFA-2013) [17] was a revision of a previous index [18], and assesses compliance with the most recent Australian Dietary Guidelines (ADG), based on current scientific evidence [3]. This analysis uses a validated tool that reflects the current guidelines using the large national survey—the National Nutrition and Physical Activity Survey-2011/2012 (NNPAS). The aim of this study is to determine the association between demographic, lifestyle and weight status with overall diet quality in Australian adults. Using a sample of the Australian population to examine the relationship between diet quality and socio-demographic factors and weight-status will determine the groups most at risk and will enable development of targeted interventions that focus on the foods and nutrients that will specifically improve diet quality of sub-groups.

## 2. Materials and Methods

### 2.1. Respondents

Data reported in this study were obtained from the Australian Bureau of Statistics (ABS) 2011–2012 National Nutrition and Physical Activity Survey (NNPAS) [19]. The survey collected nutrition and health information of the Australian population. The interview components of the NNPAS were conducted under the Census and Statistics Act (CSA) 1905. A total sample of 12,153 persons (aged 2 years and over) from 9519 private dwellings across Australia was included.

### 2.2. Dietary Data Collection

Dietary information was collected using a face-to-face 24-hour recall (24HR) interview based on the Automated Multiple-pass Method developed by the Agricultural Research Service of the United States Department of Agriculture [20] A second 24HR was conducted at least 8 days apart from the first interview via phone interview for 64% of participants from the first interview (*n* = 7735). Further information about the survey is published by the ABS [19]. This study utilised data from 9345 adults (age ≥ 18 years) who completed the first 24HR interview. Intake of nutrients from food was derived from the Australian specific nutrient composition database AUSNUT 2012, and developed specifically for this survey by the Food Standards Australia New Zealand (FSANZ) [21].

### 2.3. Anthropometry and Demographic Characteristics

Respondents’ anthropometric measurements were objectively measured in the NNPAS survey by trained interviewers; weight (in kg) was measured to the nearest 0.1 kg with digital scales and height (in cm) was measured to the nearest 0.5 cm with a stadiometer [19]. Body Mass Index (BMI) scores of the respondents were derived from their measured weight and height using Quetelet’s metric BMI (underweight BMI < 18.5 kg/m^2^, normal weight BMI 18.5–24.9 kg/m^2^, overweight BMI 25.0–29.9 kg/m^2^, obesity BMI ≥ 30.0 kg/m^2^) [22].

Demographics in this analysis included: age, grouped as 18–24, 25–34, 35–44, 45–54, 55–64, 65–74 and 75+ years, gender: male and female, socio-economic-status, which was assessed with the Socio-Economic Index of Disadvantage for Areas (SEIFA) quintiles, where the first SEIFA quintile indicates the least advantaged areas (SEIFA includes assessment of income, level of education, disability, poor English skills, employment type (labourers or unemployed), access to a motor vehicle, marital status (divorced or separated), single parent households, overcrowded living conditions, low cost housing and no home internet access [23], self-reported smoking status recorded as “Yes” or “No”, country of birth, categorised into three groups: born in Australia, born in main English-speaking countries (New Zealand, United Kingdom, Ireland, United States of America, Canada or South Africa) or born in other countries and diabetes status, defined as current and long-term diabetes mellitus (Type 1, Type 2, Gestational Diabetes, and type unknown), self-reported at the time of interview and recorded as “Yes” or “No”.

### 2.4. HEIFA-2013

The Healthy Eating Index for Australian Adults (HEIFA-2013) was adopted as a measure of diet quality that includes variety [17]. The HEIFA-2013 is a gender-specific measure of diet quality assessing adherence to the national Dietary Guidelines [17]. It includes compliance with recommended serves of core food groups and avoidance of deleterious nutrients and discretionary foods at harmful levels. The term ‘core food groups’ as used in this study refers to grains, vegetables, fruits, dairy products, and lean meat and alternatives, as described in the Australian Guide to Healthy Eating (AGHE) [24,25]. Discretionary foods are defined by the Australian Dietary Guidelines as ‘foods high in saturated fat and/or added sugars, added salt or alcohol and low in fibre’ [19], and were identified in the survey [19]. The HEIFA-2013 has previously been validated in the adult population in Australia (age range: 18–34 years, BMI range: 16–37 kg/m^2^) applied to weighed food record and food frequency questionnaire data [17].

The HEIFA’s 11-component system includes one component for each of the five core food groups, one component for discretionary choices, four components for nutrients (fatty acids, added sugar, sodium, and alcohol), and one component for water intake. Nine components (grain, vegetable, fruit, lean meat and alternatives, dairy products, discretionary choices, fatty acids, added sugar, and sodium) each contribute a maximum of 10 points to the score with two components (water and alcohol) contributing a maximum of five points each.

The core food group components for lean meats and alternatives, dairy products and discretionary food were scored for the number of serves consumed only. The grains component was scored for number of serves of grains (5 marks) and the number of serves of wholegrains (5 marks) and vegetables and fruit components were scored for the number of serves consumed (5 marks) and the number of serves of different varieties consumed (5 marks). To assess the number of serves consumed within a food group, all individually recorded food items as well as foods that were part of a mixed dish were included. Detailed measures of all individual food components were obtained by disaggregating all mixed dishes from over 3650 recipes using the AUSNUT 2011–2013 recipe file [4] and were classified under their respective core food group. For example, the amounts of oil, chicken and non-starchy vegetable components in a chicken stirfry were disaggregated and classified accordingly. The number of serves for each food group were calculated using standard serve sizes provided by the AGHE—for example, 65 g of cooked lean red meat is counted as one serve for the lean meat and alternative group [25,26].

Marks were given incrementally for specified increases in the number of serves consumed [17]. The minimum and maximum number of serves required for marks were: lean meat and alternatives: 1 to 3 serves for males, and 0.5 to 2.5 for females; dairy products: 0.5 to 2.5 serves; discretionary foods: 6 to 3 serves; grains: 1 to 6 serves; wholegrains: 1 to 3 serves; vegetables: 1 to 6 serves for males, and 1 to 5 serves for females; and fruit: 0.5 to 2 serves. The variety score was calculated for vegetables if the respondents reported consumption of at least one serve (75 g) each of the following varieties of vegetables: green, orange, cruciferous, tuber or bulb and 0.5 serves of legumes, with 1 mark given for each variety. The full 5 marks were given for fruit variety if 2 or more varieties of fruit were consumed. No marks were given for scores below the minimum ranges. For example, if a male consumed < 3 serves of discretionary food they would receive 10 marks; for 3–3.9 serves they received 7.5 marks; for 4.0–4.9 serves they received 5 marks; for 5–5.9 they received 2.5 marks; and no marks if they consumed ≥ 6 serves.

The fat component was scored for saturated fats (5 marks) and mono-unsaturated and poly-unsaturated (5 marks). Energy from saturated fat ≤10% scored 5 marks; >10%–12% scored 2.5 marks; and >12% scored 0 marks. The minimum and maximum ranges for the number of serves of poly and mono unsaturated fatty acids were 1 and 4 serves for males and 0.5 and 2 serves for females. Sodium intake <1610 mg scored 10 marks; 1610 mg to 2300 mg scored 5 marks; and >2300 mg scored 0 marks. Water intake was assessed as the proportion of water consumed in relation to other beverages with >50% scoring 5 marks and 0% = 0 marks, with each 10% increase between 0 and 50 scoring an additional 1 mark. Alcohol intake was scored as the number of standard drinks (10 g of alcohol) consumed and ≤2 standard drinks scored 5 marks and >2 standard drinks scored 0 marks. The criteria for scoring foods containing added sugars has been updated here. In April 2016, the food composition tables for Australia were updated to include “added sugars”, whereas, previously, only total sugar was available and was used in the HEIFA-2013 calculations [27]. The review of the scientific evidence to formulate the ADG suggested that added sugar should contribute no more than 10% of energy to the diet, and that a lower threshold of 5%–10% may be appropriate [3]. Percentage energy from added sugar was calculated for each respondent with <5% added sugar scoring 10 marks; 5%–10% scoring 5 points and >10% scoring 0 marks. The total maximum score for all components of the HEIFA-2013 is 100 and higher HEIFA-2013 scores indicate closer adherence to the dietary guidelines.

### 2.5. Misreporting

Misreporting of food intake is common in self-report dietary intake assessments, and 16%–26% of adult respondents in 2011–2012 NNPAS have been identified as under-reporting their total energy intake [19]. Excluding misreporters is likely to provide a more accurate interpretation and has been shown to improve the estimates obtained in similar national surveys [28,29,30]. The Goldberg cut-off method (energy intake: Basal metabolic rate 0.87–2.67) [31,32] was used to identify potential under-reporters (*n* = 1607), over-reporters (*n* = 143) and plausible reporters (*n* = 6264). No energy intake: basal metabolic rate (EI:BMR) was reported for some respondents (*n* = 1422) and therefore they could not be classified and a separate category was created for people with no recorded EI:BMR. The effect of misreporting was controlled for in the regression models used to examine associations of HEIFA with other variables. Unadjusted data are available in Supplementary Table S1.

### 2.6. Statistical Analyses

Statistical analyses were performed using SPSS for Windows 22.0 software (IBM Corp, IBM SPSS Statistics for Windows, Version 22.0, Armonk, NY, USA). Descriptive statistics were used to report the HEIFA-2013 scores according to different socio-demographic and anthropometric characteristics. Analysis of variance with post hoc analysis (Bonferroni and Tukey’s) was used to compare mean differences in total scores and to compare differences for individual components of the HEIFA-2013 scores adjusted for energy intake for each socio-demographic variable separately. Multivariate regression analysis was undertaken to investigate factors associated with the HEIFA-2013. The categorical variables including age, BMI category, gender, SEIFA, country of birth, Diabetes Mellitus status and smoking status were all entered into the model simultaneously to determine which factors were independently associated with the total score, controlling for the effects of energy (total kJ) and misreporting status (low, plausible, high and unknown). *p* < 0.05 were deemed statistically significant.

## 3. Results

The mean (SD) for the total HEIFA-2013 score for the Australian population was low at 45.5 (14.7) out of 100. Table 1 shows the mean score for the HEIFA-2013 for different subpopulations. Females scored significantly higher than males as did those in older age brackets. Those living in higher SEIFA quintiles, in the healthy or over weight-range, non-smoking, born in non-English speaking countries, or reporting a diagnosis of Diabetes Mellitus, achieved better scores than those in other sub-groups.

The mean scores for the individual components for different socio-demographic variables for the population are shown in Table 2. Low total scores were due to low individual scores for most components including discretionary food, vegetables, fruit, grain, dairy products, fat, added sugar and sodium (5 out of 10 or lower), with grain scoring the worst (lower than 3 out of 10). Higher scores were given for water and alcohol (scoring 4 out of 5).

Overall better scores were achieved for older adults compared to young adults and women compared with men due to a greater number of serves and a variety of vegetables and fruit and higher scores for water and sodium (Table 2). In addition, women achieved higher scores than men for dairy, lean meat and alcohol but lower scores for added sugar and discretionary foods when adjusted for energy. Older adults also achieved lower scores for alcohol. People within the healthy weight range scored higher mean scores than those in the obese weight-range due to higher scores for grains, fruit, discretionary food, fat and alcohol, whereas the lean meat score was significantly lower. Higher HEIFA-2013 scores were associated with older age, being female, of higher socio-economic status, born outside Australia, healthy BMI status, not smoking and self-reporting diabetes.

The multivariate analysis examining the association between change in the total score and respondents’ characteristics is shown in Table 3. The total HEIFA-2013 score was independently associated with older age and BMI (healthy vs. obese), and was higher in females and those of higher socio-economic status, not born in Australia, not smoking and those reporting diabetes.

## 4. Discussion

The overall diet quality of the Australian population is poor, with a mean diet quality score of less than half the maximum score. Poor diet quality was attributed to low consumption and poor variety of fruits, vegetables, grains and dairy, poor consumption of fats (high saturated fat intake and low polyunsaturated and mono- unsaturated fatty acids) and a high intake of sodium and alcohol. Population subgroups with lower diet quality included those from lower socio-demographic backgrounds, young adults, men, smokers, people not-reporting diabetes and those in the obese body-weight range.

The greatest disparity in diet quality of the analysed socio-demographic variables was for age, with young adults 18–24 years old consuming a diet of the lowest quality. This was due to younger age groups consuming greater amounts of sodium and added sugar while consuming less vegetables, fruit and water compared with their older counterparts. The overall less favorable diet quality of younger respondents has been consistently found in other research [33,34,35,36], and it has been suggested that age is one of the most important modifiers of diet quality [37]. Younger populations have been shown to be more likely to consume commercially prepared meals (takeaways and eating out), which displace healthier foods in the diet [38]. Australian young adults have been shown to be a group at high risk of weight gain over the past few decades [39,40]. As the poorest diet quality has been found in overweight young adults [36], diet quality may be an important point of intervention.

In our analysis, females had better diet quality than males, a finding consistent with other research [33,35,41]. These higher scores for women were due to lower intakes of sodium and alcohol, but higher scores for vegetables, fruit, dairy products, lean meat, water, and fat. Research suggests that women are more likely than men to select foods for their health benefits or for maintaining a lower body weight [42,43,44]. Men may perceive healthy eating or dieting as a feminine pursuit [45]. Accumulating evidence from weight-loss interventions suggests that programs that specifically target men are more successful at engaging men in healthy eating patterns [46]. Gender specific interventions may be required to address the gender gap in diet quality.

People with a BMI outside of the healthy weight range tended to have poorer diet quality than those outside of the healthy weight range. Consistent with a recent analysis of the NNPAS using the Diet Quality Index, a validated tool based on the AGHE, with a lower odds of obesity found for those with higher diet quality adding to the robustness that the finding here [47]. Findings on diet quality and weight status from other research have been mixed. Several cross-sectional studies have found a relationship between higher BMI and lower diet quality scores [48,49,50] and lower risk of abdominal obesity with lower diet quality [51]. Conversely, other research has found that adults with a higher BMI had better diet quality [35], and it was postulated that this might be due to misreporting, as overweight and obese participants are more likely to misreport foods than others [35]. As the effect of misreporters was controlled for in this analysis, our findings may be a better reflection of the true association between dietary intake and BMI.

People living in socio-economically disadvantaged areas had poorer diet quality scores, mostly due to lower scores for fruit, whole grains, lean meat, water, discretionary foods and added sugar and fat. Socio-economic disadvantage has been found in other research in Australia and internationally to be associated with diet quality [33,52,53]. Lower income is commonly cited as the main barrier to consuming healthier diets [54]. Internationally, healthier diets have also been found to be more expensive than unhealthy diets [54,55,56], but, in Australia, the average household spends more on food than would be required for a diet that complies with the current guidelines. The public’s perception that healthy diets are more expensive is thought to be an important barrier to healthy eating in Australia [57]. Ensuring that healthy diets become more accessible should be a priority.

The diet quality for those born in Australia and other English speaking countries including New Zealand, Canada, USA and the UK was the same. Conversely, people born outside of English-speaking countries had the highest diet quality of all subpopulations examined here. These findings are consistent with the frequent observation that the typical western diet is poor compared to other cultures and similar low scores were found for the US population, obtained with the Healthy Eating Index-2010 [58]. The higher diet quality of those born in non-English speaking countries is perhaps reflective of traditional, higher quality eating patterns that are retained by migrants to Australia; for example, African migrant children who retained their traditional values, had a lower prevalence of obesity than those that had assimilated into Australian culture [59]. Similarly, Japanese-American men living in Hawaii who had acculturated to an American diet had a higher prevalence of Type 2 Diabetes [60]. This implies that improving the quality of the western diet requires challenging cultural norms. As there has been a international shift towards western-style eating patterns [61], reversing this trend is of international significance. Younger generations are observed to be more likely to adopt new lifelong habits and it has been suggested that interventions targeting the young may be necessary to improve the diet quality of the population [61,62].

Higher diet quality index scores have been shown to be associated with reduced risk of Type 2 Diabetes [16,63] and lower incidence of Gestational Diabetes [64]. Diet quality is also an important aspect of managing Diabetes Mellitus and its associated co-morbidities. As such, diet quality indices are recommended as a tool for use in the clinical and public health setting as they encapsulate the changes necessary to improve health outcomes [16]. Participants of the survey that self-reported Diabetes Mellitus scored higher than the total population. This is presumably due to greater awareness of healthier dietary patterns, as treatment generally includes dietary advice. Recommendations vary but consistently include advice to increase vegetable intake and reduce saturated fat intake [65,66]. HEIFA-2013 component scores for added sugar, discretionary food and fruit here were higher than the total population. This is consistent with results from National Health and Nutrition Examination Survey that found poor compliance to fruits, vegetables and fat recommendations [65] for people with self-reported Type 2 diabetes. Further efforts to improve diet quality of this group are recommended.

In 2015, the World Health Organisations (WHO) released guidelines that strongly recommended that total free sugar should provide < 10% of energy due to the relationship between body weight and free sugar intake, with evidence that reductions of <5% of energy may be even more beneficial. Free sugar, in addition to sugars added during processing, extends the definition of added sugars to include intrinsic sugars ‘freed’ from their original food matrix, such as fruit juice and highly concentrated forms of sugar such as honey [67]. At the time of the review of the scientific evidence to inform the ADG-2013, the evidence that fruit juice contributed to weight gain was insufficient to make evidence-based guidelines, and it was recommended that added sugar rather than free sugar should be limited [3]. In keeping with the guidelines, scores presented here are for added sugar. The scores for the added sugar component of the population were poor indicating that a reduction in added sugar consumption is desirable but results would be worse if free sugars had been used to calculate the sugar score. Broadening the definition of added sugars to free sugar is an area of revision for future guidelines, particularly given the persistent increases in the prevalence of obesity in Australia [68].

This study analysed data from a large, nationally representative sample of the Australian population. The use of 24HR allowed people to report detailed food and beverage consumption and all food in mixed dishes were disaggregated into individual food components, which enabled a more accurate discription of food group intake. This study used a single 24HR from the survey that might not be representative of individuals’ habitual intake but was used to enable a snapshot of the population’s dietary intake. While this cross-sectional analysis does not allow for a causal relationship to be established, it indicates that lower diet quality may be a contributing factor to the high prevalence of obesity in Australia, and the effect of weight change and diet-quality warrants further investigation.

## 5. Conclusions

The overall diet quality of the population was poor, indicating low compliance with the dietary guidelines. Several socio-demographic groups had lower diet quality, including men, young adults, obesity, lower socio-economic status, smokers and English speaking country of birth. Given the substantial health benefits and reduced risk of non-communicable disease attributed to a higher quality diet, public health interventions are indicated to improve the quality of the diet of the Australian population with particular attention paid to address the needs of subpopulations with lower diet quality.

## Figures and Tables

**Table 1:** Healthy Eating Index for Australian Adults (HEIFA-2013) mean (SD) score ^1^ by socio-demographic subgroups.

		Total		Male		Female	
		N	Mean (SD)	N	Mean (SD)	N	Mean (SD)
Total Score		9435	45.5 (14.7)	4329	43.3 (14.7)	5106	47.5 (14.4)
	*p* ^						<0.001
Age (year)	18–24	780	41.6 (14.2)	373	41 (14.5)	407	42.2 (13.9)
	25–34	1617	43.7 (15) *	747	41.9 (15.2)	870	45.3 (14.6) *
	35–44	1843	44.6 (14.7)	846	42.5 (14.6)	997	46.5 (14.5)
	45–54	1660	46 (14.4)	781	43.2 (14.5)	879	48.5 (13.9) *
	55–64	1432	46.9 (14.9)	672	44 (15)	760	49.4 (14.3)
	65–74	1255	48.4 (14.5)	561	45.6 (14.6)	694	50.6 (14.1)
	75+	848	47.3 (14)	349	45.4 (14.1)	499	48.6 (13.8)
	*p* ^		<0.001		<0.001		<0.001
BMI (kg/m^2^)	<18.5	121	43.9 (15.8)	38	39.9 (14.6)	83	45.7 (16.1)
	18.5–24.9	2736	46.5 (14.9)	1059	44 (14.8)	1677	48 (14.7)
	25.0–29.9	2898	45.5 (14.9)	1659	43.7 (14.7)	1239	47.8 (14.8)
	≥30.0	2203	44.1 (14.3) *	1030	42.1 (14.5) *	1173	45.9 (13.8) *
	*p* ^		<0.001		0.004		0.001
SEIFA quintiles	1-Lowest	1778	43.7 (14.9)	796	41.2 (15)	982	45.8 (14.4)
	2	1961	44.8 (14.6)	898	42.6 (14.7)	1063	46.6 (14.2)
	3	1873	45.1 (14.4)	860	43 (14.3)	1013	46.8 (14.2)
	4	1666	46.4 (14.9)	795	44.3 (14.9)	871	48.4 (14.7)
	5-Highest	2157	47.5 (14.6)	980	44.8 (14.6)	1177	49.6 (14.2)
	*p* ^		<0.001		<0.001		<0.001
COB	Australia	6714	44.2 (14.6)	3043	41.7 (14.5)	3671	46.2 (14.4)
	English speaking ^2^	1155	45.7 (15) *	561	42.8 (14.9)	594	48.4 (14.5) *
	Others	1566	51.4 (13.7) *	725	50 (14) *	841	52.7 (13.3) *
	*p* ^		<0.001		<0.001		<0.001
Smoker	Yes	1785	40 (14.2)	931	37.9 (14.1)	854	42.3 (13.9)
	No	7650	46.8 (14.5)	3398	44.7 (14.6)	4252	48.5 (14.3)
	*p* ^		<0.001		<0.001		<0.001
Diabetes status	No	8844	45.4 (14.8)	4019	43 (14.7)	4825	47.3 (14.5)
	Yes	591	48.4 (13.8)	310	46.8 (14.5)	281	50.2 (12.7)
	*p* ^		<0.001		<0.001		0.001
Misreporting ^3^	Under-reporting	1607	47.1 (12.4)	730	45.5 (13)	877	48.4 (11.6)
	Plausible report	6263	45 (15.2) *	3017	42.8 (15)	3246	47.1 (15.2)
	Over-reporting	139	44.5 (15.5)	68	41.2 (14.9)	71	47.5 (15.6)
	No valid measurement	1426	46.2 (14.6)	514	42.8 (15)	912	48.2 (14)
	*p* ^		<0.001		<0.001		0.048

BMI: Body Mass Index; SEIFA: Socio-Economic Index for Area of disadvantage; COB: Country of Birth; ^1^ HEIFA-2013: Healthy Eating Index for Australian Adults, estimates adjusted for energy (kJ). The potential range of HEIFA-2013 score was 0–100; ^2^ English speaking countries include New Zealand, United Kingdom, Ireland, United States of America, Canada or South Africa; ^3^ Misreporting: the Goldberg cut-off method (energy intake: basal metabolic rate) was used to identify potential under-reporters (<0.87), over-reporters (>2.67) and plausible reporters (0.87–2.67); ^ *p*: Student’s *t*-test for gender, diabetes and smoking status and ANOVA analysis for differences for each group; * *p* < 0.05 from post hoc test (Bonferroni) indicates significant differences from the above category.

**Table 2:** Mean (SD) and mean differences for individual components of Healthy Eating Index for Australian Adults (HEIFA-2013) score (discretionary food, vegetable, fruit, grain, meat, dairy, water, unsaturated fat, sodium, and added sugar and alcohol scores) by demographic and socio-economic characteristics.

		Mean (SD) HEIFA-2013 Component Score ^1^										
		Grain	Veg.	Fruit	Dairy	Meat	Water	D.F.	Fat	Sodium	A.S.	Alcohol
Gender												
	Male	2.1 (0.1)	3.9 (0.2)	3.1 (0.2)	3.6 (0.2)	4.9 (0.2)	4.2 (0.1)	5 (0.2)	3.4 (0.2)	4.2 (0.2)	5.2 (0.3)	4.1 (0.1)
	Female	2.0 (0.1)	4.5 (0.2)	3.8 (0.2)	4.1 (0.2)	5.4 (0.2)	4.6 (0.1)	4.8 (0.2)	3.9 (0.2)	4.6 (0.2)	5.0 (0.2)	4.4 (0.1)
	*p* ^	0.05	<0.001	<0.001	<0.001	<0.001	<0.001	0.024	<0.001	<0.001	0.009	<0.001
Age (years)												
	18–24^R^	2 (0.3)	3.6 (0.4)	2.4 (0.6)	3.7 (0.5)	5 (0.5)	4.2 (0.2)	5.0 (0.5)	3.6 (0.4)	3.8 (0.5)	3.9 (0.6)	4.7 (0.2)
	25–34	2.1 (0.2)	4 (0.3)	3 (0.4) *	3.9 (0.3)	5 (0.4)	4.4 (0.1) *	4.9 (0.4)	3.6 (0.3)	4.2 (0.4) *	4.5 (0.4) *	4.5 (0.2) *
	35–44	2.0 (0.2)	4.1 (0.2) *	3.1 (0.4) *	4.0 (0.3)	5.2 (0.3) *	4.4 (0.1) *	4.9 (0.3)	3.7 (0.3)	4.3 (0.3) *	5 (0.4) *	4.2 (0.2) *
	45–54	2.0 (0.2)	4.1 (0.3) *	3.5 (0.4) *	3.9 (0.3)	5.3 (0.4) *	4.4 (0.1) *	5 (0.4)	3.8 (0.3)	4.5 (0.4) *	5.6 (0.4) *	4.1 (0.1) *
	55–64	2.0 (0.2)	4.4 (0.3) *	3.8 (0.4)*	3.8 (0.4)	5.4 (0.4) *	4.4 (0.1) *	4.8 (0.4)	3.8 (0.3)	4.7 (0.4) *	5.7 (0.4) *	3.9 (0.3) *
	65–74	2.1 (0.2)	4.6 (0.3) *	4.3 (0.4) *	3.8 (0.4)	5.2 (0.4)	4.6 (0.1) *	5.1 (0.4)	3.7 (0.3)	4.8 (0.4) *	5.6 (0.5) *	4.1 (0.2) *
	75+	2.2 (0.3)	4.5 (0.4) *	4.5 (0.5) *	3.9 (0.5)	4.8 (0.5) *	4.5 (0.2) *	4.9 (0.5)	3.4 (0.4)	4.9 (0.5) *	4.8 (0.6) *	4.3 (0.2) *
	*p*^	0.144	<0.001	<0.001	0.18	0.1	<0.001	0.36	0.30	<0.001	<0.001	<0.001
BMI ^2^ (kg/m^2^)												
	UW	2.1 (0.8) *	4.1 (1)	3.2 (1.4) *	4.1 (1.2)	4.5 (1.4) *	4.2 (0.4) *	4.8 (1.3) *	4.1 (1) *	4.3 (1.3)	4.1 (1.5)	4.5 (0.6) *
	Norma ^R^	2.3 (0.2)	4.4 (0.2)	3.8 (0.3)	4.1 (0.3)	5.1 (0.3)	4.5 (0.1)	5 (0.3)	3.7 (0.2)	4.6 (0.3)	5.1 (0.3)	4.3 (0.1)
	OW	2.1 (0.2) *	4.2 (0.2)	3.6 (0.3) *	3.8 (0.3) *	5.3 (0.3)	4.4 (0.1)	4.8 (0.3) *	3.7 (0.2)	4.3 (0.3)	5.2 (0.3)	4.1 (0.1) *
	Obese	1.8 (0.2) *	4.1 (0.2)	3.1 (0.3) *	3.9 (0.3)	5.5 (0.3) *	4.3 (0.1)	4.5 (0.3) *	3.5 (0.2)*	4.1 (0.3)	4.8 (0.4)	4.2 (0.1) *
	*p* ^	<0.001	0.30	<0.001	0.052	<0.001	0.06	0.001	0.002	0.10	0.22	0.001
SEIFA ^3^												
	1	1.9 (0.2) *	4.1 (0.3)	2.9 (0.4) *	3.8 (0.3)	4.9 (0.4) *	4.3 (0.1) *	4.8 (0.3) *	3.6 (0.3) *	4.4 (0.3)	4.8 (0.4) *	4.3 (0.2)
	2	2.0 (0.2) *	4.3 (0.2)	3.1 (0.3) *	3.9 (0.3)	5.2 (0.3)	4.4 (0.1) *	4.8 (0.3) *	3.5 (0.2) *	4.5 (0.3)	4.8 (0.4) *	4.3 (0.2)
	3	2.1 (0.2)	4.1 (0.2)	3.5 (0.4) *	3.9 (0.3)	5.1 (0.3)	4.4 (0.1) *	4.8 (0.3)*	3.6 (0.2) *	4.5 (0.3)	4.9 (0.4) *	4.3 (0.2)
	4	2.1 (0.2)	4.2 (0.3)	3.9 (0.4)	3.9 (0.3)	5.2 (0.4)	4.5 (0.1)	5.1 (0.4)	3.6 (0.3) *	4.4 (0.4)	5.3 (0.4) *	4.2 (0.2)
	5 ^R^	2.2 (0.2)	4.3 (0.2)	4.0 (0.3)	4.0 (0.3)	5.3 (0.3)	4.5 (0.1)	5.1 (0.3)	3.8 (0.2)	4.4 (0.3)	5.6 (0.4)	4.2 (0.1)
	*p* ^	<0.001	0.075	<0.001	0.556	0.004	<0.001	0.001	0.004	0.878	<0.001	0.216
COB												
	Australia ^R^	1.9 (0.1)	4.1 (0.1)	3.3 (0.2)	4.0 (0.2)	5.1 (0.2)	4.4 (0.1)	4.6 (0.2)	3.5 (0.1)	4.3 (0.2)	4.8 (0.2)	4.2 (0.1)
	English ^4^	2.0 (0.2)	4.2 (0.3)	3.7 (0.5) *	4.0 (0.4)	5.0 (0.4)	4.5 (0.1)	4.8 (0.4) *	3.6 (0.3)	4.6 (0.4) *	5.3 (0.5) *	4.0 (0.2) *
	Others	2.8 (0.2) *	4.4 (0.3) *	4.1 (0.4) *	3.3 (0.3) *	5.4 (0.4)	4.6 (0.1) *	6.4 (0.4) *	4.4 (0.3) *	5.1 (0.4) *	6.0 (0.4) *	4.6 (0.2) *
	*p*^	<0.001	<0.001	<0.001	<0.001	0.80	<0.001	<0.001	<0.001	<0.001	<0.001	<0.001
Diabetes Mellitus												
	No	2 (0.1)	4.2 (0.1)	3.4 (0.2)	3.9 (0.1)	5.2 (0.2)	4.4 (0.1)	4.9 (0.2)	3.7 (0.1)	4.5 (0.2)	5 (0.2)	4.2 (0.1)
	Yes	2.2 (0.4)	4.3 (0.4)	4 (0.6)	3.8 (0.6)	5.3 (0.6)	4.4 (0.2)	5.3 (0.6)	3.5 (0.4)	4.4 (0.6)	6.2 (0.7)	4.3 (0.3)
	*p* ^	0.057	0.247	0.001	0.64	0.359	0.67	0.006	0.334	0.683	<0.001	0.18

Veg: Vegetables, D.F. Discretionary foods, A.S.: Added sugars, R: Referent, BMI: Body Mass Index.; UW: Under-weight, OW: Over-weight, SEIFA: Socio-Economic Index for Area of disadvantage, COB: Country of Birth; ^1^ Mean scores adjusted for energy. The potential range of HEIFA-2013 score was 0–100, water and alcohol were 0–5, and the rest were 0–10; ^2^ For BMI, the cut offs for underweight, normal weight, overweight and obesity were <18.5 kg/m^2^, 18.5–24.9 kg/m^2^, 25.0–29.9 kg/m^2^ and ≥30.0kg/m^2^, respectively; ^3^ SEIFA quintiles: 1 is the lowest and 5 is the highest; ^4^ English: English speaking countries including New Zealand, United Kingdom, Ireland, United States of America, Canada or South Africa; ^ *p*: Student’s *t*-test for gender and Diabetes Mellitus, ANOVA for age, BMI, SEIFA and COB.; * *p* < 0.05 from post hoc test (Bonferroni) indicates significant difference from the referent.

**Table 3:** Association of HEIFA-2013 score with socio-demographic covariates.

HEIFA Score		β	SE	*p*
Gender (Ref. Male)	Female	3.2	0.3	<0.001
Age (Ref. 18–24)	25–34	1.9	0.6	0.002
	35–44	3.0	0.6	<0.001
	45–54	4.3	0.6	<0.001
	55–64	4.9	0.6	<0.001
	65–74	5.4	0.7	<0.001
	75+	3.6	0.7	<0.001
BMI ^2^ (Ref. Healthy weight)	Underweight	−1.9	1.3	0.139
	Overweight	−0.6	0.3	0.1
	Obesity	−2.7	0.4	<0.001
Country of birth (Ref. Australia)	English-speaking ^3^	1.1	0.4	0.015
	Others	6.5	0.4	<0.001
SEIFA Quintiles (Ref. 1st)	2^nd^	<0.001	<0.001	0.99
	3^rd^	<0.001	<0.001	0.99
	4^th^	<0.001	<0.001	0.98
	5th-highest	0.7	0.4	0.045
Smoking (Ref. No smoker)	-6.0	0.4	<0.001	Smoking (Ref. No smoker)
Diabetes (Ref. No)	2.1	0.6	0.001	Diabetes (Ref. No)

Scores adjusted for the effects of energy-reporting status and total energy (kJ); Ref.: referent, β indicates the change in HEIFA score per unit change of the covariates; SE: Standard Error; BMI: Body Mass Index; SEIFA: Socio-Economic Index for Area of disadvantage.

## Supplementary Materials

The following are available online at www.mdpi.com/2227-9032/5/1/7/s1. Table S1: Mean (SD) for individual components of Healthy Eating Index for Australian Adults (HEIFA-2013) score (discretionary food, vegetable, fruit, grain, meat, dairy, water, fat, sodium, and added sugar and alcohol scores) for demographic and socio-economic characteristics for all respondents including under- and over-reporters.
