# Supplementary material for: Socio-Demographic Determinants of Diet Quality in Australian Adults Using the Validated Healthy Eating Index for Australian Adults (HEIFA-2013)
Source: Healthcare (Basel). 2017 Feb 4;5(1):7. doi: 10.3390/healthcare5010007 (PMC5371913; doi:10.3390/healthcare5010007)
Supplement: Supplementary file 1 [file healthcare-05-00007-s001.pdf]

Supplementary Materials: Socio-Demographic Determinants of Diet Quality in Australian Adults Using the Validated Healthy Eating Index for Australian Adults (HEIFA-2013)

Amanda Grech, Zhixian Sui, Hong Ying Siu, Miaobing Zheng, Margaret Allman-Farinelli and Anna Rangan

Table S1. Mean (SD) for individual components of Healthy Eating Index for Australian Adults (HEIFA-2013) score (discretionary food, vegetable, fruit, grain, meat, dairy, water, unsaturated fat, sodium, and added sugar and alcohol scores) for demographic and socio-economic characteristics for all respondents of the National Nutrition and Physical Activity Survey–2011/2012.

Score	Mean (SD)	Grain	Veg.	Fruit	Dairy	Meat	Water	D.F.	Fat	Sodium	A.S.	Alcohol
**Gender**												
	Male	2.4 (2.4)	4.1 (2.7)	3.3 (4)	4 (3.7)	5.4 (4.1)	4.2 (1.5)	4.2 (4.3)	3.4 (2.6)	3.4 (4.1)	5.0 (4.3)	3.9 (2.1)
	Female	1.8 (2.2)	4.3 (2.8)	3.6 (4)	3.8 (3.5)	5 (3.8)	4.6 (1)	5.5 (4.2)	3.9 (2.8)	5.3 (4.3)	5.1 (4.3)	4.5 (1.5)
**Age (years)**												
	18–24	2.2 (2.2)	3.7 (2.6)	2.5 (3.6)	3.9 (3.8)	5.2 (4)	4.2 (1.5)	4.5 (4.3)	3.6 (2.7)	3.4 (4.2)	3.8 (4.2)	4.6 (1.4)
	25–34	2.3 (2.3)	4.1 (2.7)	3.1 (3.8)	4.1 (3.7)	5.2 (4)	4.3 (1.4)	4.5 (4.3)	3.6 (2.7)	3.8 (4.2)	4.4 (4.3)	4.4 (1.6)
	35–44	2.1 (2.2)	4.1 (2.7)	3.1 (3.9)	4.1 (3.6)	5.3 (4)	4.4 (1.3)	4.7 (4.3)	3.7 (2.8)	4.1 (4.3)	4.9 (4.3)	4.1 (1.9)
	45–54	2 (2.3)	4.1 (2.8)	3.5 (4)	3.9 (3.6)	5.4 (4)	4.4 (1.2)	4.9 (4.3)	3.8 (2.7)	4.4 (4.4)	5.6 (4.3)	4.1 (1.9)
	55–64	1.9 (2.3)	4.4 (2.9)	3.8 (4)	3.7 (3.5)	5.3 (4)	4.5 (1.2)	5 (4.3)	3.8 (2.7)	4.9 (4.3)	5.7 (4.2)	4 (2)
	65–74	2 (2.4)	4.5 (2.8)	4.2 (4.1)	3.6 (3.4)	5 (4)	4.6 (1)	5.6 (4.2)	3.7 (2.7)	5.3 (4.3)	5.7 (4.2)	4.2 (1.8)
	75+	1.9 (2.4)	4.4 (2.8)	4.3 (4)	3.5 (3.4)	4.4 (3.8)	4.6 (1)	5.7 (4.1)	3.4 (2.8)	5.7 (4.2)	5 (4.2)	4.5 (1.5)
**BMI**												
	<18.5	2.1 (2.4)	4.1 (2.9)	3.2 (4)	4.2 (3.9)	4.6 (3.8)	4.2 (1.6)	4.7 (4.5)	4.1 (2.9)	4.2 (4.5)	4.1 (4.3)	4.5 (1.6)
	18.5–24.9	2.3 (2.4)	4.4 (2.8)	3.8 (4.1)	4.1 (3.7)	5.1 (4)	4.5 (1.2)	4.8 (4.3)	3.7 (2.8)	4.4 (4.3)	5 (4.3)	4.2 (1.8)
	25.0–29.9	2.2 (2.4)	4.2 (2.7)	3.6 (4)	3.9 (3.6)	5.3 (4)	4.4 (1.3)	4.7 (4.3)	3.7 (2.7)	4.2 (4.3)	5.2 (4.2)	4.1 (1.9)
	≥30.0	1.7 (2)	4.1 (2.8)	3.1 (3.8)	3.8 (3.6)	5.4 (4)	4.4 (1.3)	4.8 (4.3)	3.5 (2.7)	4.4 (4.4)	4.8 (4.3)	4.3 (1.8)
**SEIFA**												
	1-Lowest	1.8 (2.2)	4.0 (2.8)	2.8 (3.8)	3.7 (3.6)	4.8 (4)	4.3 (1.4)	4.9 (4.3)	3.6 (2.8)	4.6 (4.4)	4.8 (4.4)	4.3 (1.7)
	2	1.9 (2.3)	4.2 (2.8)	3 (3.8)	3.8 (3.7)	5.2 (4)	4.4 (1.3)	4.9 (4.3)	3.5 (2.6)	4.6 (4.3)	4.8 (4.3)	4.3 (1.8)
	3	2.1 (2.3)	4.2 (2.7)	3.5 (4)	3.9 (3.6)	5.2 (4)	4.4 (1.3)	4.7 (4.3)	3.6 (2.8)	4.4 (4.4)	4.8 (4.2)	4.2 (1.8)
	4	2.1 (2.3)	4.2 (2.7)	3.9 (4.1)	3.9 (3.5)	5.3 (4)	4.5 (1.2)	5.1 (4.3)	3.6 (2.8)	4.3 (4.3)	5.3 (4.2)	4.2 (1.8)
	5-Highest	2.3 (2.4)	4.3 (2.7)	4 (4.2)	4 (3.6)	5.4 (4)	4.5 (1.1)	5 (4.3)	3.8 (2.7)	4.4 (4.3)	5.6 (4.2)	4.1 (1.9)
**COB**												
	Australia	1.9 (2.2)	4.2 (2.8)	3.3 (3.9)	4 (3.6)	5.2 (4)	4.4 (1.3)	4.6 (4.3)	3.5 (2.7)	4.2 (4.3)	4.8 (4.3)	4.2 (1.9)
	English	2 (2.3)	4.2 (2.8)	3.7 (4.1)	4.1 (3.6)	5.1 (4)	4.4 (1.2)	4.8 (4.3)	3.6 (2.8)	4.5 (4.3)	5.3 (4.2)	4 (2)
	Others	2.8 (2.5)	4.4 (2.7)	4.1 (4.1)	3.2 (3.5)	5.3 (4)	4.7 (1)	6.6 (4)	4.4 (2.7)	5.3 (4.4)	6.1 (4.2)	4.7 (1.3)

Veg: Vegetables, D.F. Discretionary foods, A.S.: Added sugars, R: Referent, BMI: Body Mass Index., UW: Under-weight, OW: Over-weight, SEIFA: Socio-Economic Index for Area of disadvantage, COB: Country of Birth; [1] English: English speaking countries including New Zealand, United Kingdom, Ireland, United States of America, Canada or South Africa.
